# Protective Spinel Coating for Li_1.17_Ni_0.17_Mn_0.50_Co_0.17_O_2_ Cathode for Li-Ion Batteries through Single-Source Precursor Approach

**DOI:** 10.3390/nano10091870

**Published:** 2020-09-18

**Authors:** Andrey Shevtsov, Haixiang Han, Anatolii Morozov, Jesse C. Carozza, Aleksandra A. Savina, Iaroslava Shakhova, Nellie R. Khasanova, Evgeny V. Antipov, Evgeny V. Dikarev, Artem M. Abakumov

**Affiliations:** 1 Center for Energy Science and Technology, Skolkovo Institute of Science and Technology, Nobel str. 3, 143026 Moscow, Russia; Anatolii.Morozov@skoltech.ru (A.M.); a.savina@skoltech.ru (A.A.S.); Y.Shakhova@skoltech.ru (I.S.); antipov@icr.chem.msu.ru (E.V.A.); A.Abakumov@skoltech.ru (A.M.A.); 2 Department of Chemistry, Lomonosov Moscow State University, 119991 Moscow, Russia; nellie@icr.chem.msu.ru; 3 Department of Chemistry, University at Albany, Albany, NY 12222, USA; hx.han@cornell.edu (H.H.); jcarozza@albany.edu (J.C.C.); edikarev@albany.edu (E.V.D.); 4 Department of Materials Science and Engineering, Cornell University, Ithaca, NY 14850, USA

**Keywords:** cathode for Li-ion battery, Li-rich NMC, core–shell, protective layer, capacity fade, voltage fade

## Abstract

The Li_1.17_Ni_0.17_Mn_0.50_Co_0.17_O_2_ Li-rich NMC positive electrode (cathode) for lithium-ion batteries has been coated with nanocrystals of the LiMn_1.5_Co_0.5_O_4_ high-voltage spinel cathode material. The coating was applied through a single-source precursor approach by a deposition of the molecular precursor LiMn_1.5_Co_0.5_(thd)_5_ (thd = 2,2,6,6-tetramethyl-3,5-heptanedionate) dissolved in diethyl ether, followed by thermal decomposition at 400 °C inair resulting in a chemically homogeneous cubic spinel. The structure and chemical composition of the coatings, deposited on the model SiO_2_ spheres and Li-rich NMC crystallites, were analyzed using powder X-ray diffraction, electron diffraction, high angle annular dark-field scanning transmission electron microscopy (HAADF-STEM), and energy-dispersive X-ray (EDX) mapping. The coated material containing 12 wt.% of spinel demonstrates a significantly improved first cycle Coulombic efficiency of 92% with a high first cycle discharge capacity of 290 mAhg^−1^. The coating also improves the capacity and voltage retention monitored over 25 galvanostatic charge–discharge cycles, although a complete suppression of the capacity and voltage fade is not achieved.

## 1. Introduction

Future development of the lithium-ion battery technology is associated withthe commercial deployment of advanced positive electrode (cathode) materials, such as lithium-rich/manganese-rich *x*Li_2_MnO_3_–(1-*x*)LiMO_2_ (M = Mn, Ni, Co) or Li_4/3-*x*_Ni^2+^*_x_*Mn^4+^_2/3-*x*_Co^3+^*_x_*O_2_ (also termed as Li-rich NMC) oxides with a layered structure. Being structurally similar to LiCoO_2_, these materials demonstrate higher reversible capacity exceeding 250 mAhg^−1^ [1,2], originating from the Ni^2+^→Ni^3+,4+^ and Co^3+^→Co^4+^ cationic redox reactions and the significant contribution of the reversible anionic redox processes (2O^2−^→ O_2_*^n^*^−^, where 3 > *n* > 1) above the potential of 4.5 V vs. Li/Li^+^ [3,4,5,6,7,8,9,10]. However, the materials suffer from the very low Coulombic efficiency of the first charge–discharge cycle as well as from the substantial capacity and voltage fade (i.e., a reduction in average cell voltage) on subsequent cycles, largely preventing their broad commercialization [11]. The capacity and voltage fade are generally associated with gradually accumulating structural changes upon prolonged charge–discharge cycling. It was demonstrated that the voltage decay in such systems is closely related to the migration of cations between the Mlayers and Lilayers during the charge–discharge process that causes trapping of the M cations in the interstitial tetrahedral sites [12,13,14,15]. Such cation migration, where the transition metal cations move to the Li sites, finally transforms the layered structure to that of a spineltype [16,17]. However, the most dramatic structural changes occur at the surface of the cathode crystals, where disordered regions are formed. This disordered surface layer also irreversibly loses oxygen and some lithium that causes its “densification”, which is at the origin of the irreversible capacity at the first charge–discharge cycle. As a result, the surface of the Li-rich NMC crystals departs from the layered structure toward a disordered rock–salt structure [18,19,20,21,22]. The formation of the surface Li-depleted disordered layer in Li_4/3-*x*_Ni^2+^*_x_*Mn^4+^_2/3-*x*_Co^3+^*_x_*O_2_ (Li-rich NMC) appears particularly discouraging as it lacks diffusion channels and should therefore slow down the Li extraction/insertion. The latter further compromises the initially poor electrode kinetics and makes the disordered materials kinetically sluggish [10,23,24,25,26]. The disordered surface layer between the electrode and electrolyte can be formed after exposing the layered NMC material to the electrolyte, even without an applied potential [27]. Progressive growth of the “densified” surface layer may also contribute to the capacity fade. 

The surface coating of the Li-rich NMC cathode particles is viewed as a promising approach toward the physical separation of the cathode and electrolyte, thus potentially diminishing the aforementioned detrimental effects (although coatings might be less effective against the voltage fade [28], as the cation migration occurs in the bulk and seems to be an intrinsic and inseparable part of the charge–discharge processes). Metal oxides such as Al_2_O_3_, TiO_2_, ZrO_2_, and CeO_2_ have been used for the coatings [28,29,30,31]. However, most of these materials are poor electronic and Li-ion conductors, leading to increased Ohmic loss that can only be mitigated by lowering the thickness of the coating. A more advanced approach involves Li-conducting solid electrolytes, e.g., by applying LiTaO_3_, Li_2_ZrO_3_, Li_2_TiO_3_, or LiPON layers. The capacity retention and rate capability of Li-rich NMC at high voltages up to 4.8–4.9 V were greatly improved with the lithium tantalate or LiPON surface coatings [32,33]. It was also documented that 1 wt.% Li_2_ZrO_3_ coating stabilizes the crystal structure, decreases the oxygen loss, and enhances the thermal stability of the electrode charged to 4.8 V [34]. However, as these coatings are electronically insulating, increasing the coating thickness may cause deterioration of the electrochemical performance due to the increasing electronic resistance [33].

Instead of utilizing electrochemically inert and electronically non-conducting oxides, we propose to coat Li-rich NMC with a nanosized layer of another cathode material, thus creating core–shell architectures that demonstrate reversible Li (de)intercalation and are stable toward electrolytes at the potentials upwards of ≈5 V vs. Li/Li^+^. Spinel-type Li- and Mn-based oxides appear as plausible candidates for such coating due to their high electronic and Li^+^ ion conductivities, good rate capability, safety, and structural similarity to the substrate [35,36,37,38,39,40]. Such a protective shell will also contribute to the total capacity of the composite system, in contrast to the electrochemically inert binary oxides and Li-ion solid electrolytes. However, the chemical composition of the spinel coatings is quite complex and might comprise up to four chemical elements, making the proposed coating deposition a non-trivial procedure.

The previous studies have shown that spinel surface modification might enhance the electrochemical performance of the core material. The methods of applying the spinel coating may vary from co-precipitation via carbonates [41] to the surface treatment of Li-rich NMC with acid [42,43] or with an oxidizing agent [44]. The carbonate co-precipitation consists of applying the precursor of the protective coating onto the precursor of the core material. Although very appealing, its main shortcoming comes from the fact that it is possible to apply the surface modification only to the materials with the same structure and similar lithium content, which is not the case for the core–shell Li-rich NMC–spinel composite. The surface treatments have previously been reported to decrease the first-cycle capacity loss at the cost of poorer capacity retention [45].

In this contribution, we describe the application of a protective spinel coating to the already prepared Li-rich NMC cathode material through heterometallic volatile and soluble single-source precursors. Single-source precursors are the molecules containing all the necessary elements in the proper ratio and decomposable in a controllable manner under mild conditions to yield phase-pure materials [46]. This helps to obtain homogeneous spinel layers without any detrimental effect on the Li-rich NMC core. The reason that we did not employ a mixture of molecular (multi-source) precursors to prepare the coatings is that it might result in inhomogeneous chemical composition [47].

We have previously reported on the isolation of heterometallic diketonate LiMn_2_(thd)_5_ (thd = 2,2,6,6-tetramethyl-3,5-heptanedionate) as the first single-source precursor for the low-temperature preparation of LiMn_2_O_4_ spinel cathode material [48]. Importantly, it was recently shown that the partial substitution of manganese for other transition metals in the latter molecule is possible, thus opening broad opportunities for the design of hetero*tri*metallic precursors for the LiMn_2-*x*_M*_x_*O_4_ cathode materials [49]. Here, the LiMn_1.5_Co_0.5_O_4_ spinel was chosen as the coating material due to its voltage stability window [39,50]. Although among the LiMn_2-*x*_M*_x_*O_4_ (M = Fe, Co, Ni) spinels, the Ni-substituted one has been reported as having higher capacity retention [51], this difference is not directly inherent in higher electrochemical stability; instead, it is merely attributed to a formation of passivation surface layers at the M = Fe, Co spinels due to electrolyte decomposition at a higher upper voltage threshold (5.3V vs. Li/Li^+^ for M = Fe, Co vs. 5.1V for M = Ni) [52], where the Li-rich NMC materials usually do not operate. Here, we describe the coating of the Li_1.17_Ni_0.17_Mn_0.50_Co_0.17_O_2_ cathode material with the LiMn_1.5_Co_0.5_O_4_ spinel obtained by thermal decomposition of the LiMn_1.5_Co_0.5_(thd)_5_ molecular precursor and the electrochemical properties of the obtained core–shell structure.

## 2. Materials and Methods 

### 2.1. Preparation of the Samples

#### 2.1.1. Synthesis of the Li-Rich NMC Core Cathode Material

The synthesis of Li_1.17_Ni_0.17_Mn_0.50_Co_0.17_O_2_hasbeen carried out *via* co-precipitation of a carbonate precursor. The solution of manganese (II), nickel (II), and cobalt (II) acetates (Sigma Aldrich > 99% (St, Louis, MO, USA), Alfa Aesar > 99% (Ward Hill, MA, USA) and RusKhim > 99% (Moscow, Russia) respectively) was drop-by-drop added to the stirred solution of sodium carbonate in a stoichiometric amount in a buffer solution of NH_3_ at 75 °C. The pH was kept at 7.5 during the synthesis. The obtained pink carbonate precursor was aged for several hours, centrifuged (Liston, Zhukov, Russia), and dried in air. Solid lithium carbonate (Sigma Aldrich > 99% (St, Louis, MO, USA)) with 15% excess was added to the obtained precursor, and the mixture was ball-milled for 4 h. The reactants were first calcined at 400 °C for 2 h, ball-milled again, and finally heated at 800 °C for 4 h, resulting in black powder. 

#### 2.1.2. Synthesis of the Coated Samples

Molecular precursor LiMn_1.5_Co_0.5_(thd)_5_ (thd = 2,2,6,6-tetramethyl-3,5-heptanedionate) was used for the low-temperature deposition of the LiMn_1.5_Co_0.5_O_4_ spinel coating. The details onthe synthesis and structural investigation of precursor have already been reported [43]. High volatility and solubility of LiMn_1.5_Co_0.5_(thd)_5_ in aprotic solvents allowed us to employ two approaches for the coating deposition: (i) metal–organic chemical vapor deposition (MOCVD) (see Appendix A) and (ii) solution-based coating coupled with thermal decomposition. 

In the solution-based coating approach, the substrate was mixed with the precursor solution in diethyl ether and stirred for 10 min. After solvent evaporation, the solid residue was placed in the oven, which was set to 350–450 °C for 30 min. The resulting solid was again treated with the precursor solution, and such a procedure was repeated 10 to 25 times in different experiments. Finally, the solid was annealed in air at 400–450 °C for 12 h. All preliminary coating experiments were performed using 99.9% pure SiO_2_ spherical particles with the size of 0.5 micron as a substate. The best experimental conditions found for the SiO_2_ substrate have been used in coating experiments on Li_1.17_Ni_0.17_Mn_0.50_Co_0.17_O_2_. Two substrate/precursor mass ratios of 1:0.2 (low concentration) and 1:0.6 (high concentration) were tested for the SiO_2_ nanospheres. For the Li_1.17_Ni_0.17_Mn_0.50_Co_0.17_O_2_ substrate, three mass ratios of 1:0.2 (low concentration), 1:0.4 (medium concentration), and 1:0.6 (high concentration) were examined.

### 2.2. Characterization of Materials

The specific surface area (SSA) of the samples was measured by nitrogen adsorption using a NovaTouch device (Quantachrome, Corporate Dr, Boynton Beach, FL, USA). The determination of SSA was carried out using the NovaTouch software package (21 CFR Part 11 version of TouchWin software) based on the linear adsorption isotherm according to the theory of Brunauer, Emmett, and Teller (BET method).

The quantitative phase composition of the samples was characterized using powder X-ray diffraction (PXRD; Huber Guinier camera G670 with an ImagePlate detector, CuK_α1_ radiation Rimsting, Germany). The pattern fitting and Rietveld refinement were performed using the Jana2006 software (Prague, Czech Republic, Version string 25/10/2015) [53].

Scanning electron microscopy images were taken with a JEOL JSM-6490 LV (Eching b. München, Germany) scanning electron microscope at 30 kV accelerating voltage.

Samples for transmission electron microscopy (TEM) were prepared by dispersing materials in an agate mortar with ethanol and depositing a few drops of suspension onto copper grids covered by a holey carbon layer. Electron diffraction (ED) patterns, high angle annular dark-field scanning transmission electron microscopy (HAADF-STEM) images, and energy-dispersive X-ray (EDX) spectra were obtained with an FEI Osiris electron microscope (Hillsboro, OR, USA) operated at 200 kV using a Super-X EDX system. High-resolution TEM (HRTEM) images were acquiredwith an FEI Tecnai G^2^ F20 (Hillsboro, OR, USA) transmission electron microscope operated at 200 kV. HAADF-STEM and HR TEM images were treated using TIA (version 4.7 SP3 by FEI company (now Thermo Fisher Scientific (Waltham, MA, USA) and/or Digital Micrograph(TM) (version 1.8178 by Gatan inc., (Pleasanton, CA, USA) software packages while elemental maps were proceeded in ESPIRIT 2.0 program (Bruker company, Billerica, MA, USA).

### 2.3. Electrochemical Measurements on the Pristine and Coated Samples

The electrochemical measurements were performed in coin-type cells vs. metallic Li as anode in 1M LiPF_6_ in the 1:1 ethylene carbonate (EC)/dimethyl carbonate (DMC) electrolyte. The cells were assembled in an Ar-filled glove box. First, 12.5 wt.% of SP carbon and 12.5 wt.% of polyvinylidene fluoride (PVDF) were added to the cathode material to improve its conductive and adhesive properties. The cyclic voltammetry was performed in the 2.0–4.8V potential range. The cells were also cycled in a galvanostatic regime in the same potential range at 18 mAg^−^^1^ current density.

## 3. Results

### 3.1. Synthesis of the Li-Rich NMC Core Material

**Figure 1:**
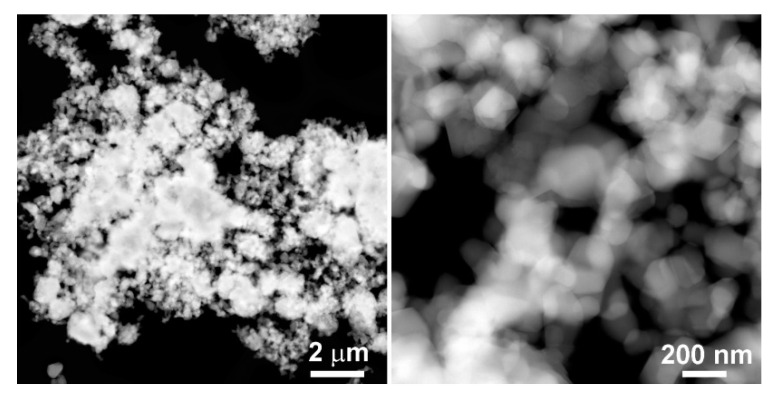
High angle annular dark-field scanning transmission electron microscopy (HAADF-STEM) images of the pristine Li_1.17_Ni_0.17_Mn_0.5_Co_0.17_O_2_ sample demonstrating highly agglomerated crystallites.

According to the PXRD pattern, the pristine Li_1.17_Ni_0.17_Mn_0.50_Co_0.17by_O_2_ sample represents a single phase. The Rietveld refinement converged to the monoclinic *C*2/*m* structure with the unit cell parameters *a* = 4.9327(9) Å, *b* = 8.576(2) Å, *c* = 5.0116(8) Å, and *β* = 109.34(2)º (Figure S1 of Supporting information). HAADF-STEM and SEM images of the pristine sample revealed that it is built of highly agglomerated crystallites of 100–200 nm in size, forming a porous network and round-shaped secondary particles (Figure 1, Figure S2). This is corroborated by the BET measurements (Figure S3), which show the specific surface area (SSA) of the material of 7.25 m^2^/g.

### 3.2. Coating of the SiO_2_Core

**Figure 2:**
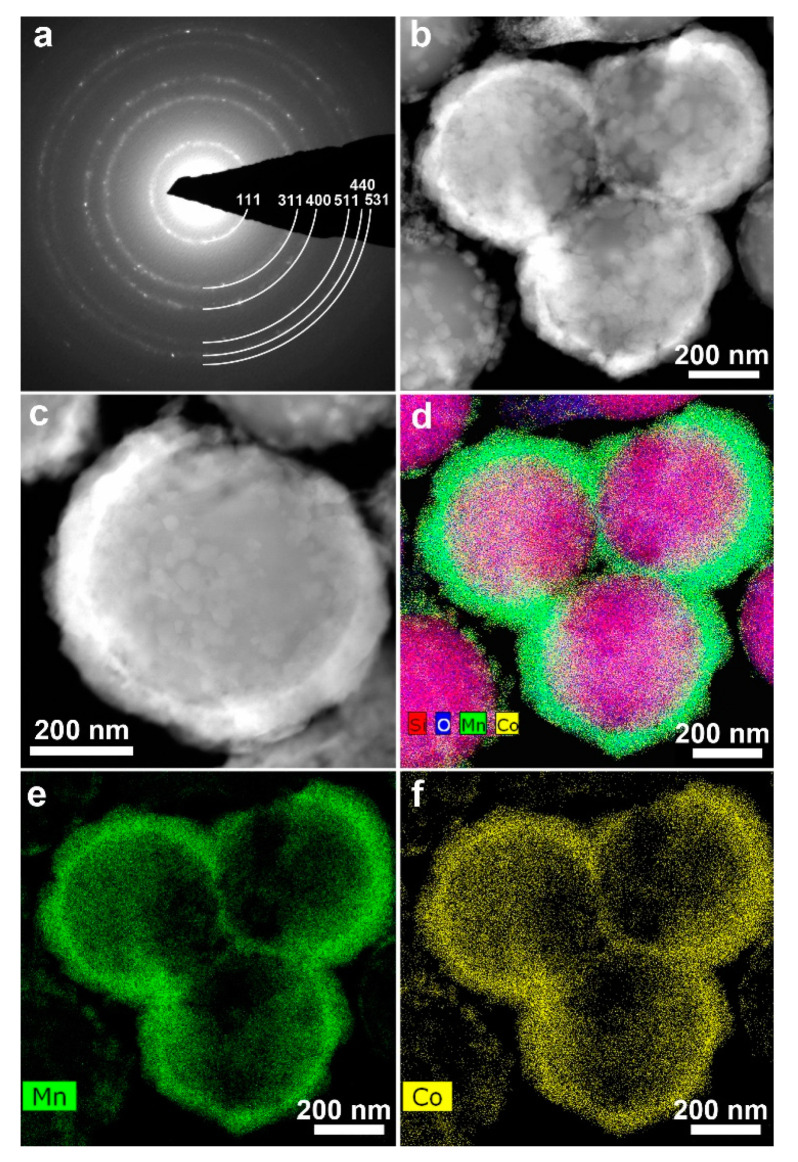
(**a**) Electron diffraction pattern of the solution-coated SiO_2_ spheres indexed with the *Fd*-3*m* spinel structure; (**b**,**c**) HAADF-STEM images showing *ca.* 100 nm spinel coating layer around the SiO_2_ spheres; (**d**) energy-dispersive X-ray (EDX) map of the Mn (green), Co (yellow), O (blue), and Si (red) distribution. (**e**,**f**) individual EDX maps of Mn and Co.

The low-concentration precursor solution did not yield a spinel coating, resulting in separate nanocrystals at the SiO_2_ surface. When the high concentration solution was applied followed by precursor decomposition at 450 °C, ring electron diffraction (Figure 2a) demonstrates that most of the SiO_2_ spheres were covered with a*ca.* 100 nm thick layer of the 10–20 nm spinel nanocrystals (Figure 2b,c). Rietveld refinement from PXRD data (Figure S4) confirms the presence of a spinel phase with the unit cell parameter *a* = 8.1677(9) Å. EDX analysis revealed the Co/Mn atomic ratio of 0.50(5):1.50(5) corresponding to the LiMn_1.5_Co_0.5_O_4_ phase; the Mn and Co distribution appeared to be homogeneous (Figure 2d–f, Figure S5). Therefore, this approach was further implemented for coating the Li-rich NMC samples.

### 3.3. Coating of the Li-Rich NMC Core

Coating conditions, crystallographic data, phase, and elemental compositions of the spinel-coated Li_1.17_Ni_0.17_Mn_0.50_Co_0.17_O_2_ samples are summarized in Table 1 and Table S1.

Rietveld refinement (Figure S6) indicateshigh content of the spinel phase in the sample annealed at 350 °C (sample I from Table 1 and Table S1). However, such low annealing temperature does not result in a complete decomposition of the precursor. According to the HAADF-STEM images and compositional EDX maps, micron-sized flakes containing Mn, Co, and C with embedded spinel nanocrystals are intermixed with the Li-rich NMC crystallites (Figure S7). The sample annealed at a higher temperature of 450 °C (sample V from Table S1) contains only very minor spinel content (≈3 wt.%) (Figure S8). The strong influence of the decomposition temperature on the final spinel content reflects that the coating process occurs at the conditions that are far from thermodynamic equilibrium. The phase composition and microstructure of the samples appear as a trade-off between the completeness of precursor decomposition, its volatilization, and the crystallinity of the obtained spinel phase.

**Figure 3:**
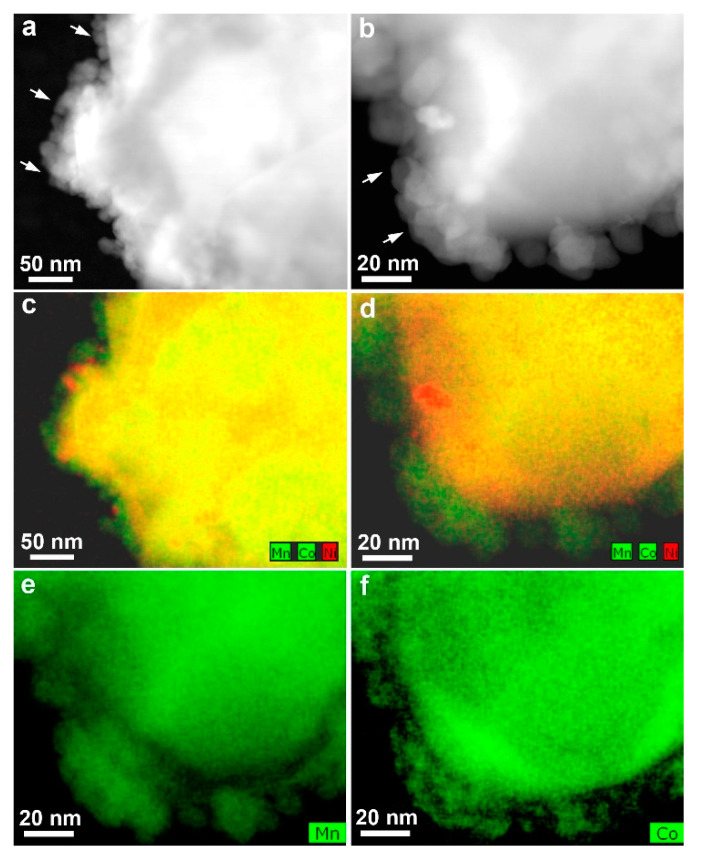
HAADF-STEM images (**a**,**b**) and compositional EDX maps (**c**,**d**) demonstrating the spinel nanocrystals covering crystallites of the Li_1.17_Ni_0.17_Mn_0.50_Co_0.17_O_2_ phase in sample II annealed at 400 °C using low precursor concentration. The spinel phase is seen as green nanocrystals at the surface as it does not contain Ni (**e**,**f**). Few Ni-rich areas (red) are attributed to local inhomogeneity in the Li-rich NMC crystals.

The most satisfactory results were obtained with the intermediate precursor decomposition temperature of 400 °C (samples II, III, and IV). Increasing the precursor concentration results in increasing the content of the spinel phase in the samples from 12 wt.% at low precursor concentration (sample II) to 23–24 wt.% at medium and high precursor concentrations (samples III and IV) (Figure S9, Table S1). For the latter samples, the HAADF-STEM images and EDX compositional maps demonstrate coverage of the Li-rich NMC faceted crystallites with spinel nanocrystals of 5–20 nm in size (Figure 3). Similar surface coverage has been observed for samples III and IV, but in these samples, prepared with higher precursor concentrations, spinel is also present as a separate phase (Figure S10).

**Figure 4:**
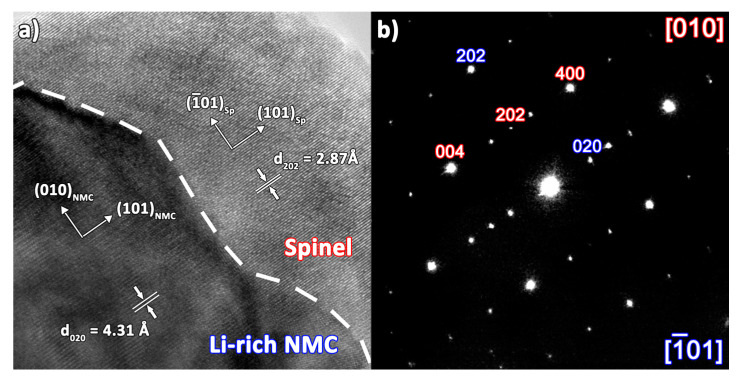
(**a**) HRTEM image of the surface of Li_4/3-*x*_Ni^2+^*_x_*Mn^4+^_2/3-*x*_Co^3+^*_x_*O_2_ (Li-rich NMC) particle covered with the LiMn_1.5_Co_0.5_O_4_ spinel layer. The dashed line marks the boundary between the layered phase and spinel. The directions in the crystal lattice for each phase are indicated by arrows. Major interplanar distances are marked. (**b**) Electron diffraction(ED) pattern corresponding to the area shown in the panel (**a**). The reflections are indexed as belonging to the layered phase (blue) and spinel (red) structures, the zone axes are also identified and given in corresponding colors.

Figure 4a shows the HRTEM image of the surface layer at the Li-rich NMC particle. The external layer has a thickness of 10–20 nm, being characterized by the interplanar spacing of 2.87Å, which is attributed to (202) crystal planes of the cubic spinel structure (sp. gr. *Fm*-3*m*, *a* ≈ 8.12 Å). The interplanar spacing of the interior part is 4.31Å, which corresponds to (020) crystal planes of Li-rich NMC (sp. gr. *C*2/*m*, *a* ≈ 4.95 Å, *b* ≈ 8.56 Å, *c* ≈ 5.03 Å, β ≈ 109.3°). The ED pattern (Figure 4b) collected from this region (Figure 4a) confirms the co-existence of the Li-rich NMC and spinel structures. In addition, both HRTEM and ED demonstrate that the spinel layer on the surface of the layered oxide grows in a way that (101101)_spinel_ || (101101)_NMC_ and (1$\bar{1}01$01)_spinel_ || (010010)_NMC_, thus forming an epitaxial structure. These results support the hypothesis of high structural compatibility between the layered phase and the spinel structure, thanks to the oxygen cubic close packing intrinsic in both structures.

### 3.4. Electrochemical Measurements on the Pristine and Coated Li-Rich NMCs

**Figure 5:**
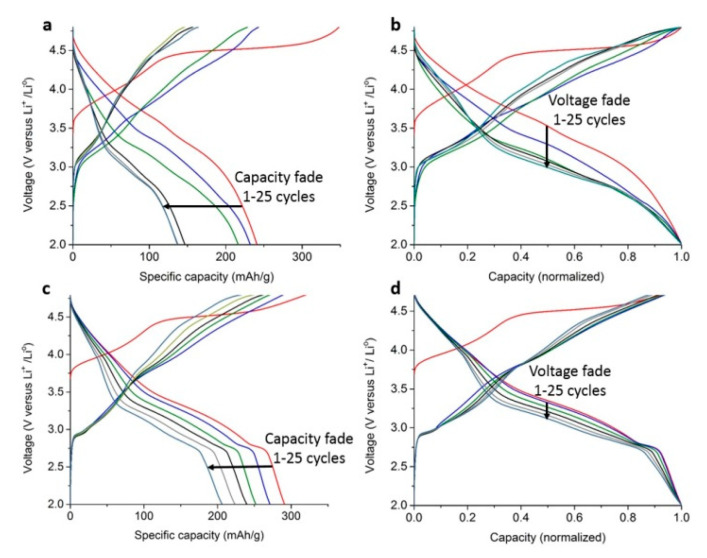
Voltage *vs.* specific capacity plots cycled at 18 mAg^−^^1^current density for pristine Li_1.17_Ni_0.17_Mn_0.50_Co_0.17_O_2_ (**a**) and ≈12 wt.% spinel-coated sample II (**c**); voltage *vs.* normalized capacity plots (maximum capacity in each cycle is taken as unity) for pristine Li_1.17_Ni_0.17_Mn_0.50_Co_0.17_O_2_ (**b**) and sample II (**d**).

The cyclic voltammetry of the pristine material and sample II shows the electrochemical activity between 2 and 4.8 V (Figures S11 and S12). Sample II shows higher performance in the upper voltage range. The galvanostatic cycling of pristine Li-rich NMC demonstrates the behavior typical for this class of cathode materials. The first charge occurs through two distinct stages: the sloping region in the 3.5–4.4 V potential range corresponding to the cationic redox on Ni^2+^ and Co^3+^and the long plateau at ≈4.5 V originating from anionic redox and leading to the total capacity of 345 mAhg^−^^1^ (Figure 5a). The discharge capacity on the first charge–discharge cycle amounts to 240 mAhg^−^^1^ only, indicating low Coulombic efficiency of 70%. After the first cycle, the discharge curves turn to a well-known S-type shape. The following cycles demonstrate significant capacity loss from 240 to 130 mAhg^−^^1^. In addition to the capacity fade, a very pronounced voltage fade on discharge is visible after 25 charge–discharge cycles, manifesting itself in flattening of the discharge profiles, resulting in a reduction of the average cell voltage by ≈500 mV. Sample II has been selected for a more detailed study because of its electrochemical properties (see Appendix B for the electrochemical properties of other samples) and the most homogeneous shell distribution.

The initial charge capacity of sample II, coated with ≈12 wt.% of spinel at 400 °C, is 315 mAhg^−^^1^ (Figure 5c). The first charge capacity is largely retained on first discharge (290 mAhg^−^^1^), providing the Coulombic efficiency of 92%. Further galvanostatic cycling reveals much better capacity retention of 71% over the first 25 cycles compared to *ca.* 50% for the pristine material (Figure 5c). The discharge curves demonstrate a characteristic short plateau at ≈2.8 V that is usually associated with the reversible first-order cubic-to-tetragonal spinel transformation [54]. The normalized galvanostatic curves (where the maximum capacity in each cycle is taken as unity) were used to compare the voltage decay in pristine Li-rich NMC and the coated sample II. Coating with the Mn-Co spinel significantly suppresses the voltage decay (Figure 5b,d). The PXRD pattern of sample II after 25 charge–discharge cycles demonstrates a presence of both spinel and Li-rich NMC phases, indicating the stability of the spinel coating (Figure S14). The comparative study of the electrochemical performance of the pristine material versus the coated sample as a function of the current rate ranging from 18 to 180 mAg^−^^1^ clearly shows, especially at higher rates, the advantage of using a spinel protective layer (Figure S15). 

However, further increase of the thickness of the protective coating does not improve the capacity and voltage retention. The first cycle capacity of sample III with ≈24 wt.% spinel at 400 °C (Figure S15a) with a less homogeneous protective coating has a lower discharge capacity (265 mAhg^−^^1^) compared to sample II, which is probably due to the higher percentage of the spinel phase present in the sample. The first cycle Coulombic efficiency is 99%. The spinel coating negates the initial cycle capacity loss. Yet, starting from cycle 5, the Coulombic efficiency decreases significantly, resulting in high overcharges, which in turn lead to faster degradation of the material and voltage fade (Figure S15b).The PXRD pattern of sample III after 25 charge–discharge cycles shows the broadening of the Li-rich NMC phase peaks with no possibility to identify two separate phases (Figure S16). The protective spinel coating proved to be too thick for the optimum performance of the core material.

## 4. Discussion

The successful strategy of an Li-rich NMC particles coating with a crystalline and chemically homogeneous spinel layer has utilized the advantages of single-source precursors over the multi-source precursors based on homometallic complexes: (i) homogeneity at the molecular level that diminishes diffusion problems in the following reaction steps; (ii) easier stoichiometry control that helps to avoid the formation of off-stoichiometric products; (iii) overcoming differences in the thermal behavior and chemical incompatibility of the multiple precursor complexes; (iv) the formation of decomposition products at significantly lower temperatures (400–450 °C in our work); and (v) flexibility in the preparation of functional materials, allowing deposition of the material through either CVD (chemical vapor depostion) or solution-based routes. Unfortunately, a directcomparisonofthe single- andmulti-source precursor is not possible. The physical mixture of three separate components—Li(thd), Mn(thd)_2_ and Co(thd)_2_—would not resultin a singular compound, as Mn(thd)_2_ and Co(thd)_2_ do not have the same solubility. On top of that, Li(thd) is insoluble in the solvent we used in the study, and homometallic precursor, Mn(thd)_2_, is extremely air-sensitive to the point that it is nearly impossible to use it for the preparation of the desired spinel. Therefore, only by incorporating three metals into a single molecule could all the issues be solved and the material synthesized.

The temperature of the precursor decomposition is a key factor determining the quality of the obtained coating, and reasonable compromise should be found to minimize the precursor loss due to its volatility, maximize the decomposition degree, and achieve good crystallinity of the spinel phase. In the reported case, thermal decomposition of the heterometallic precursor at 400 °C provided the best result.

The comparative galvanostatic cycling of the pristine and spinel-coated Li-rich NMC materials demonstrates a noticeable improvement of Coulombic efficiency at the first charge–discharge cycle, as well as better capacity and voltage retention during the subsequent cycles. The large drop of capacity on first discharge represents a well-known disadvantage of the Li-rich NMC layered oxides. It is commonly accepted that low Coulombic efficiency at the first charge–discharge is associated with the compositional and structural changes occurring at the 4.5 V plateau. This plateau is responsible for an extra capacity beyond that provided by purely cationic Ni^2+^/Ni^3+,4+^and Co^3+^/Co^4+^ redox processes. Above 4.5 V, the oxygen sublattice becomes an active participant of the redox reaction, delivering extra electrons taken from non-bonding (also called “orphaned”) O2*p* states localized on the oxygen atoms involved into the Li-O-Li chains due to partial replacement of the transition metal cations in the (Li,M)O_3_ layers with Li [55]. Partial oxygen oxidation might be reversible, causing covalent O–O bonding along with a formation of peroxo-like species in the Li-rich layered oxides of the 4*d* and 5*d* transition metals [3,5,56]. However, for the 3*d* transition metal oxides, a competing oxygen decoordination leads to irreversible gaseous oxygen evolution, which is also accompanied by the loss of Li^+^, a partial reduction of Mn^4+^ to Mn^3+^, and “densification” of the surface layer with a formation of spinel-like or rock salt-like structures [20,21,57,58]. Direct contact with the electrolyte plays a key role in cathode degradation during the first charge–discharge cycle. The reactions at the cathode/electrolyte interface are very complex and not fully understood. It is believed that oxygenevolving from the cathode surface at the potentials > 4.5 V oxidizes the carbonate solvent producing CO_2_, CO, and H_2_O, which in turn react with LiPF_6_, releasing HF [59,60]. Acidic media promotes Li dissolution accompanied by a migration of the transition metal cations toward the octahedral Li positions ending up with a disordered rock–salt structure (“densification”). Once formed, the “densified” layer protects the remaining Li-rich layered material from further oxygen release, prevents contact with the electrolyte, and acts as a protecting cathode–electrolyte interface. Indeed, the first cycle Coulombic efficiency can be enhanced by a reductive surface treatment of the Li-rich layered oxides causing a pre-formation of the spinel protective layer [61,62]. In our case, the deposited protective spinel coating greatly improves the first cycle Coulombic efficiency by effectively separating the surface of the Li-rich NMC cathode from direct contact with the electrolyte. The LiMn_1.5_Co_0.5_O_4_ spinel belongs to the 5 V class of cathode materials and is known to be stable toward oxygen evolution. There is a certain similarity between the electrochemical behavior of the spinel-coated Li-rich NMC oxide and the so-called “spinel-layered” composites, where the spinel domains are structurally embedded into the layered matrix or even form a physical two-phase mixture. In these composites, the first cycle capacity fade also decreases upon introducing the spinel component [63,64,65,66]. 

Although it seems that the spinel coating mitigates oxygen evolution and detrimental effects of electrolyte oxidation, it is not possible to fully suppress the capacity and voltage fade in the subsequent cycles. Oxygen redox promotes migration of the transition metal cations to the octahedral Li positions, causing nucleation of the spinel-like domains near the surface area with subsequent propagation toward the interior of the Li-rich NMC crystallites [67,68,69]. This transformation manifests itself as the *S*-shaped discharge curve due to a spectrum of energetically different cationic and oxygen positions. The degree of voltage decay correlates with the length of the oxygen redox plateau, emphasizing that power loss due to the gradually decreasing average voltage seems to be an unavoidable price for the extra capacity gained through anion redox [70]. Nevertheless, the significant voltage fade dumping observed in the spinel-coated samples reveals that the coating indirectly affects the transition metal cation migration, either diminishing the fraction of the migrated cations or increasing the migration reversibility. Improved capacity retention appears as another positive consequence of the suppressed cation migration, following the suggested mechanism of a mechanical detachment of the spinel nanocrystals due to increasing microstrain between the layered matrix and spinel nanodomains [68,71]. One can speculate that the partial prevention of oxygen evolution with the protective spinel coating slows down the nucleation of the spinel-like nanodomains, which improves the capacity and voltage retention over prolonged cycling, but it cannot fully eliminate the capacity and voltage fade.

## 5. Conclusions

A protective LiMn_1.5_Co_0.5_O_4_ spinel coating has been applied to the Li_1.17_Ni_0.17_Mn_0.50_Co_0.17_O_2_ (Li-rich NMC) cathode material using a single-source precursor approach. Solution-based deposition and low-temperature decomposition of the molecular precursor LiMn_1.5_Co_0.5_(thd)_5_ produced spinel nanocrystals covering the surface of the Li-rich NMC crystallites. The resulting core–shell structure, in which a layer of a high-voltage spinel cathode material prevents direct contact of the Li-rich NMC surface with the electrolyte, demonstrates improved electrochemical performance compared to that of the pristine uncoated material. The first cycle irreversible capacity is largely eliminated, resulting in 92% Coulombic efficiency while maintaining high first discharge capacity of 290 mAhg^−^^1^. The coated Li-rich NMC sample also demonstrates the improved capacity and voltage retention monitored over 25 galvanostatic charge–discharge cycles. 

## 6. Patents

Based on the work, a patent has been granted. Abakumov A.M., Dikarev E.V., Han H., and Shevtsov A., Skolkovo Institute of Science and Technology, “Protective spinel coatings for Ni-Mn-Co (NMC) cathode with increased Li content for Li-ion batteries, application method of the aforementioned coating on the cathode and the cathode with the aforementioned coating”, RU 2 702 785 C1, 2019.

## Figures and Tables

**Table 1:** Precursor concentration [c] and decomposition temperature of the coated Li_1.17_Ni_0.17_Mn_0.50_Co_0.17_O_2_ samples.

Sample	I	II	III	IV	V
Conditions	Medium [c] 350 °C	Low [c] 400 °C	Medium [c] 400 °C	High [c] 400 °C	Low [c] 450 °C

**Figure A1:**
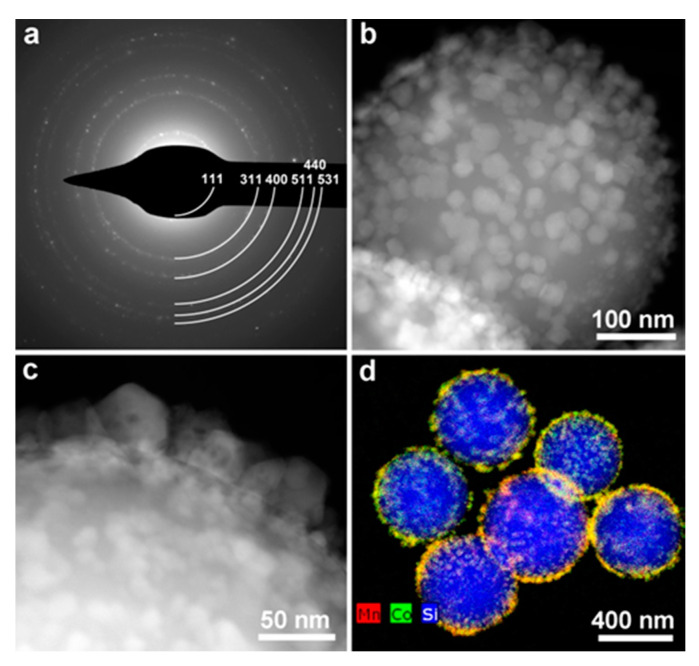
(**a**) Electron diffraction pattern of the metal–organic chemical vapor deposition (MOCVD)-coated SiO_2_ spheres indexed with *Fd*-3*m* spinel structure; (**b**,**c**) HAADF-STEM images showing the spinel nanocrystals at the surface of the SiO_2_ spheres; (**d**) EDX map of the Mn (red), Co (green), and Si (blue) distribution.

## Supplementary Materials

The following are available online at https://www.mdpi.com/2079-4991/10/9/1870/s1, Figure S1. Experimental, calculated and difference PXRD profiles after the Rietveld refinement of pristine Li_1.17_Ni_0.17_Mn_0.5_Co_0.17_O_2_. The bars mark the reflection positions for the monoclinic C2/m structure, Figure S2. SEM images of the pristine Li_1.17_Ni_0.17_Mn_0.5_Co_0.17_O_2_, Figure S3. Results of BET measurements, Figure S4. Experimental, calculated and difference PXRD profiles after the Rietveld refinement of the spinel phase in the solution-coated SiO_2_ spheres. The bars mark the reflection positions for the cubic spinel structure, Figure S5. The profiles of the Mn, Co, Si and O EDX signals across a SiO2 sphere coated with the spinel layer. Note homogeneous distribution of Mn and Co, Figure S6. Experimental, calculated and difference PXRD profiles after the Rietveld refinement of the spinel (top reflection row) and Li_1.17_Ni_0.17_Mn_0.5_Co_0.17_O_2_ (bottom reflection row) phases in the sample **I**, Figure S7. HAADF-STEM image and elemental EDX maps for Mn, Co, Ni and C for the sample I. The Ni-containing areas are the Li-rich NMC crystallites, Figure S8. Experimental, calculated and difference PXRD profiles after the Rietveld refinement of the spinel (top reflection row) and Li_1.17_Ni_0.17_Mn_0.5_Co_0.17_O_2_ (bottom reflection row) phases in the sample **V**, Figure S9. Experimental, calculated and difference PXRD profiles after the Rietveld refinement of the spinel (top reflection row) and Li_1.17_Ni_0.17_Mn_0.5_Co_0.17_O_2_ (bottom reflection row) phases in the samples **II** (a), **III** (b) and **IV** (c), Figure S10. Cyclic voltammetry of the pristine material in the 2.0–4.8 V potential range, Figure S11. Cyclic voltammetry of the sample II in the 2.0–4.8 V potential range, Figure S12. HAADF-STEM images (top) and compositional EDX maps (bottom) demonstrating a presence of spinel as a separate phase (left column) and as nanocrystals covering crystallites of the Li_1.17_Ni_0.17_Mn_0.5_Co_0.17_O_2_ phase (right column) for the sample **III** annealed at 400 °C with medium precursor concentration. The spinel phase is seen as green areas and nanocrystals at the surface as it does not contain Ni. The Ni-containing areas (red, yellow) are the Li-rich NMC crystals, Figure S13. Experimental, calculated and difference PXRD profiles after the Rietveld refinement of the spinel (top reflection row) and Li_1.17_Ni_0.17_Mn_0.5_Co_0.17_O_2_ (bottom reflection row) phases in the samples **II** after 25 galvanostatic cycles at C/20, Figure S14. Rate performance of the pristine material (black) and coated (red), Figure S15. Voltage *vs.* specific capacity plots for the ~24 wt.% spinel-coated sample **III**(a). Voltage *vs.* normalized capacity plots (maximum capacity in each cycle is taken as a unity) for sample **III** (b), Figure S16. Experimental, calculated and difference PXRD profiles after the leBail refinement of the Li1.17Ni0.17Mn0.5Co0.17O2 phase in the samples III after 25 galvanostatic cycles at C/20, Table S1. Precursor concentration, decomposition temperature, unit cell parameters, phase weight fractions, and the results of EDX analysis of the coated Li_1.17_Ni_0.17_Mn_0.5_Co_0.17_O_2_ samples. The core material Li-rich NMC has the monoclinic structure (*C*2/*m*) and the protective spinel coating LiMn_1.5_ Co_0.5_O_4_ was identified as a cubic crystal (*Fd*-3*m*).

## Appendix A. Coating Deposition via Metal–Organic Chemical Vapor Deposition (MOCVD)

In the MOCVD experiments, the precursor was deposited in a horizontal CVD reactor *via* evaporation in Ar flow (gas pressure of ≈ 200 mTorr) onto the substrate surface at continuous stirring in air. The precursor/substrate mass ratio was 1:10, and the deposition was repeated five times. The precursor evaporation temperature was varied from 150 to 350 °C in different experiments, while the decomposition temperature ranged between 450 and 650 °C.

First coating experiments with the CVD technique on the model SiO_2_ spheres readily demonstrated the feasibility of the selected strategy to form a homogeneous mixed-transition metal spinel phase *via* decomposition of the LiMn_1.5_Co_0.5_(thd)_5_ single-source precursor (300 °C evaporation temperature, 650 °C decomposition temperature, 1:10 precursor/substrate ratio, five cycles of deposition process). The ring electron diffraction pattern of the coated spheres was indexed with a cubic *Fd*-3*m* symmetry with the unit cell parameter *a* ≈ 8.2 Å, confirming a formation of the spinel phase (Figure A1a). The surface of the SiO_2_ spheres was found to be coated with the plate-like spinel nanocrystals (Figure 3b,c) featuring a homogeneous distribution of Mn and Co cations (Figure A1d). However, the coverage of the surface by spinel crystals was found to be incomplete with many crystals growing in the unwanted “edge-on” mode (Figure A1c). Moreover, the MOCVD method appeared to be ineffective, resulting in excessive consumption of the precursor. Thus, the following investigations were focused on the alternative method, the solution-based deposition technique (see the main text).

## Appendix B. Comments on the Electrochemical Performance for Samples I, III, IV, and V

The electrochemical performance of sample V annealed at 450 °C is identical to the pristine Li-rich NMC that can be attributed to a very low fraction of the spinel phase in this sample (2.7 wt.%). Samples I (medium concentration, 350 °C) and IV (high concentration, 400 °C) could not be properly distributed on the surface of the aluminum current collector, resulting in electrodes of poor quality compared to the pristine material. This is likely related to the appearance of a large fraction of the semi-amorphous secondary phase due to incomplete precursor decomposition in sample I and the presence of a separate nanocrystalline spinel phase in sample IV. Sample III reaches high initial capacity; however, it degrades at a rate similar to the pristine material, alongside the voltage, due to the non-homogenous distribution of the protective spinel shell (Figure S12).
