# Supplementary material for: Protective Spinel Coating for Li1.17Ni0.17Mn0.50Co0.17O2 Cathode for Li-Ion Batteries through Single-Source Precursor Approach
Source: Nanomaterials (Basel). 2020 Sep 18;10(9):1870. doi: 10.3390/nano10091870 (PMC7558323; doi:10.3390/nano10091870)
Supplement: Supplementary file 1 [file nanomaterials-10-01870-s001.pdf]

Supplementary Materials

Protective Spinel Coating for $Li_{1.17}Ni_{0.17}Mn_{0.50}Co_{0.17}O_2$ Cathode for Li-Ion Batteries through Single-Source Precursor Approach

Andrey Shevtsov [1,2,*], Haixiang Han [3,4], Anatolii Morozov [1,2], Jesse C. Carozza [3], Aleksandra A. Savina [1], Iaroslava Shakhova [1], Nellie R. Khasanova [2], Evgeny V. Antipov [1,2], Evgeny V. Dikarev [3] and Artem M. Abakumov [1]

[1] Center for Energy Science and Technology, Skolkovo Institute of Science and Technology, Nobel str. 3, Moscow 143026, Russia; Anatolii.Morozov@skoltech.ru (A.M.), a.savina@skoltech.ru (A.A.S.), Y.Shakhova@skoltech.ru (I.S.), A.Abakumov@skoltech.ru (A.M.A.)

[2] Department of Chemistry, Lomonosov Moscow State University, Moscow 119991, Russia; nellie@icr.chem.msu.ru (N.R.K.), antipov@icr.chem.msu.ru (E.V.A.)

[3] Department of Chemistry, University at Albany, Albany, New York 12222, United States; jcarozza@albany.edu (J.C.C.), edikarev@albany.edu (E.V.D.)

[4] Department of Materials Science and Engineering, Cornell University, New York 14850, United States; hx.han@cornell.edu (H.H)

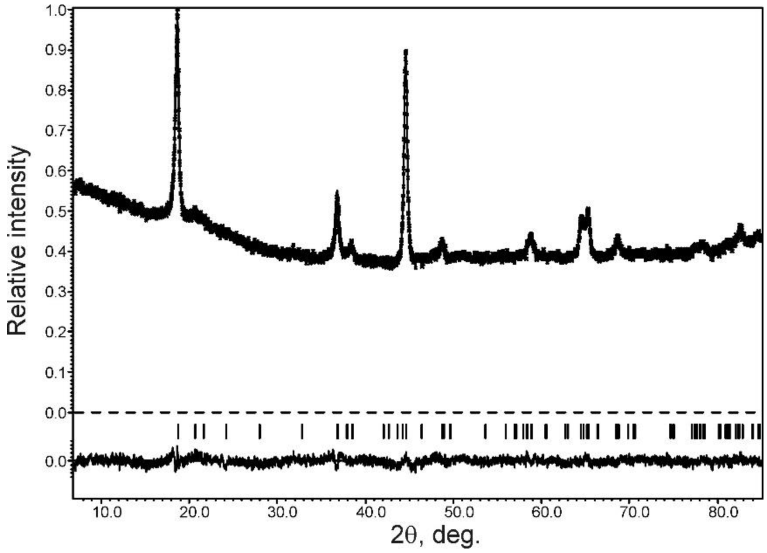

Figure S1. Experimental, calculated and difference PXRD profiles after the Rietveld refinement of pristine $Li_{1.17}Ni_{0.17}Mn_{0.5}Co_{0.17}O_2$. The bars mark the reflection positions for the monoclinic *C*2/*m* structure.

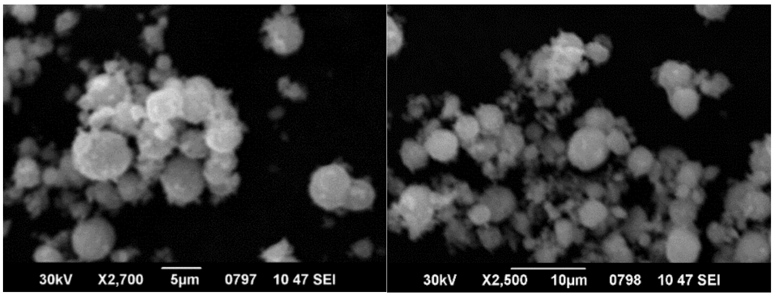

Figure S2. SEM images of the pristine $Li_{1.17}Ni_{0.17}Mn_{0.5}Co_{0.17}O_2$.

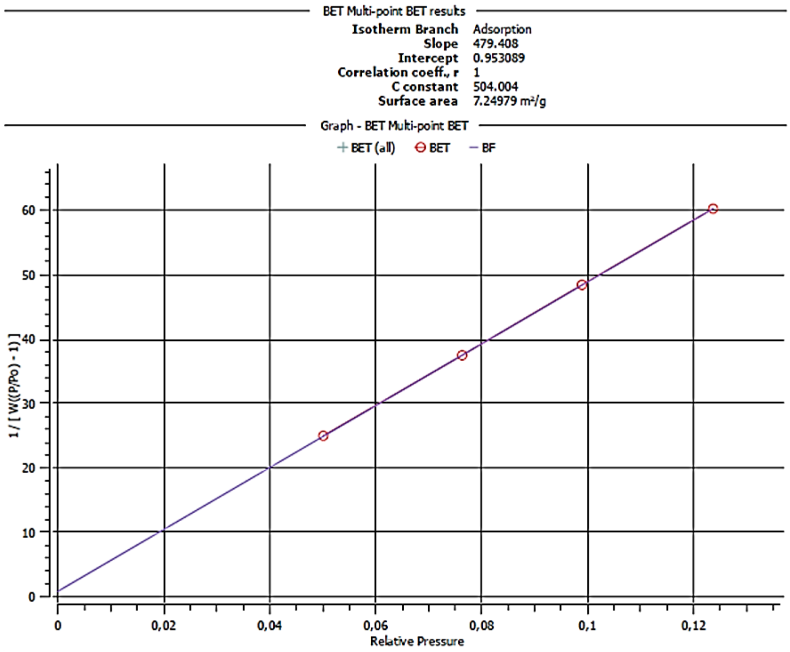

Figure S3. Results of BET measurements.

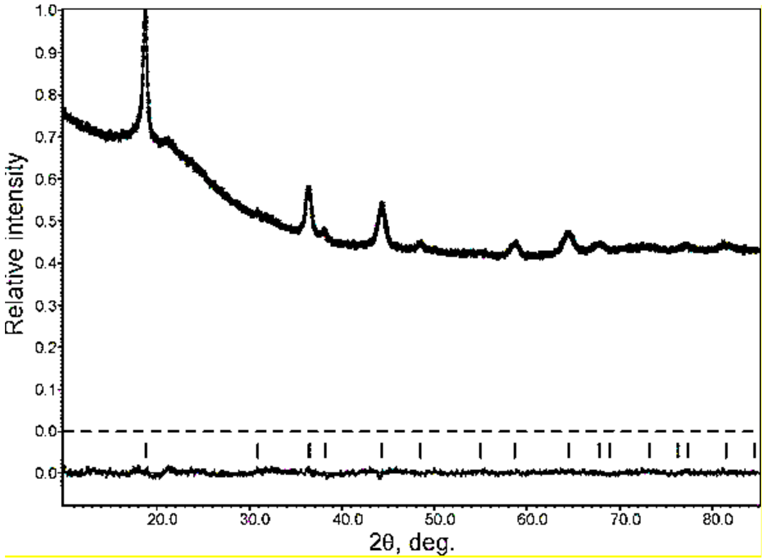

Figure S4. Experimental, calculated and difference PXRD profiles after the Rietveld refinement of the spinel phase in the solution-coated SiO_2 spheres. The bars mark the reflection positions for the cubic spinel structure.

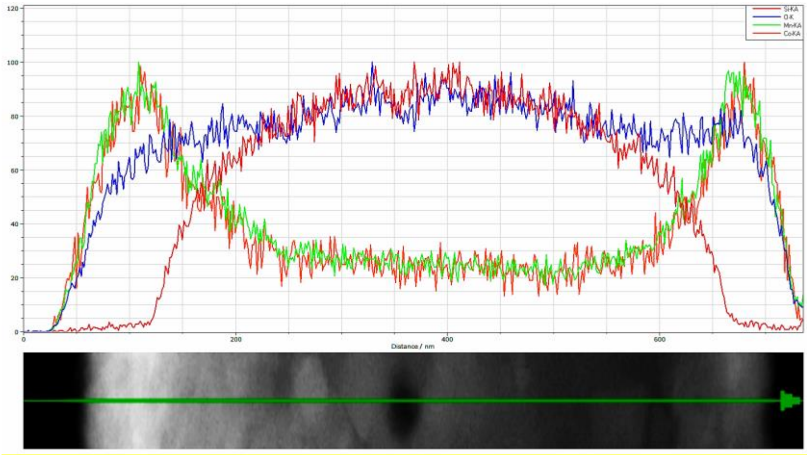

Figure S5. The profiles of the Mn, Co, Si and O EDX signals across a SiO_2 sphere coated with the spinel layer. Note homogeneous distribution of Mn and Co.

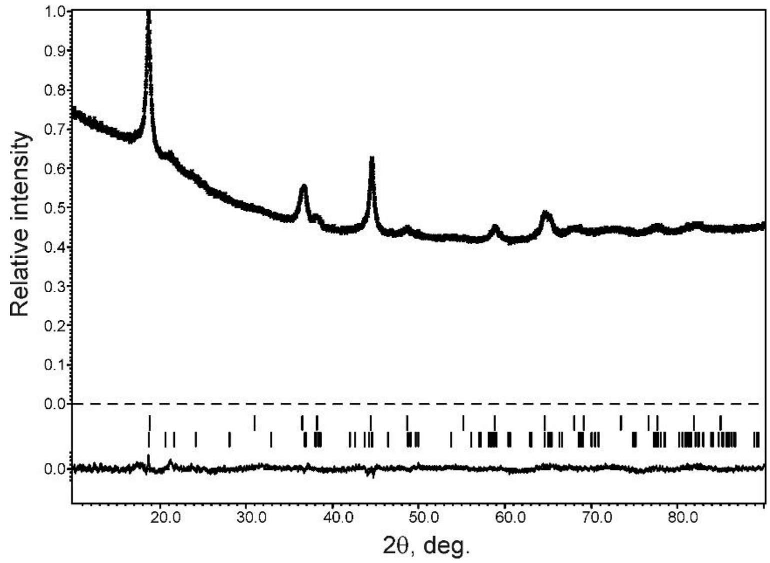

Figure S6. Experimental, calculated and difference PXRD profiles after the Rietveld refinement of the spinel (top reflection row) and $Li_{1.17}Ni_{0.17}Mn_{0.5}Co_{0.17}O_2$ (bottom reflection row) phases in the sample **I**.

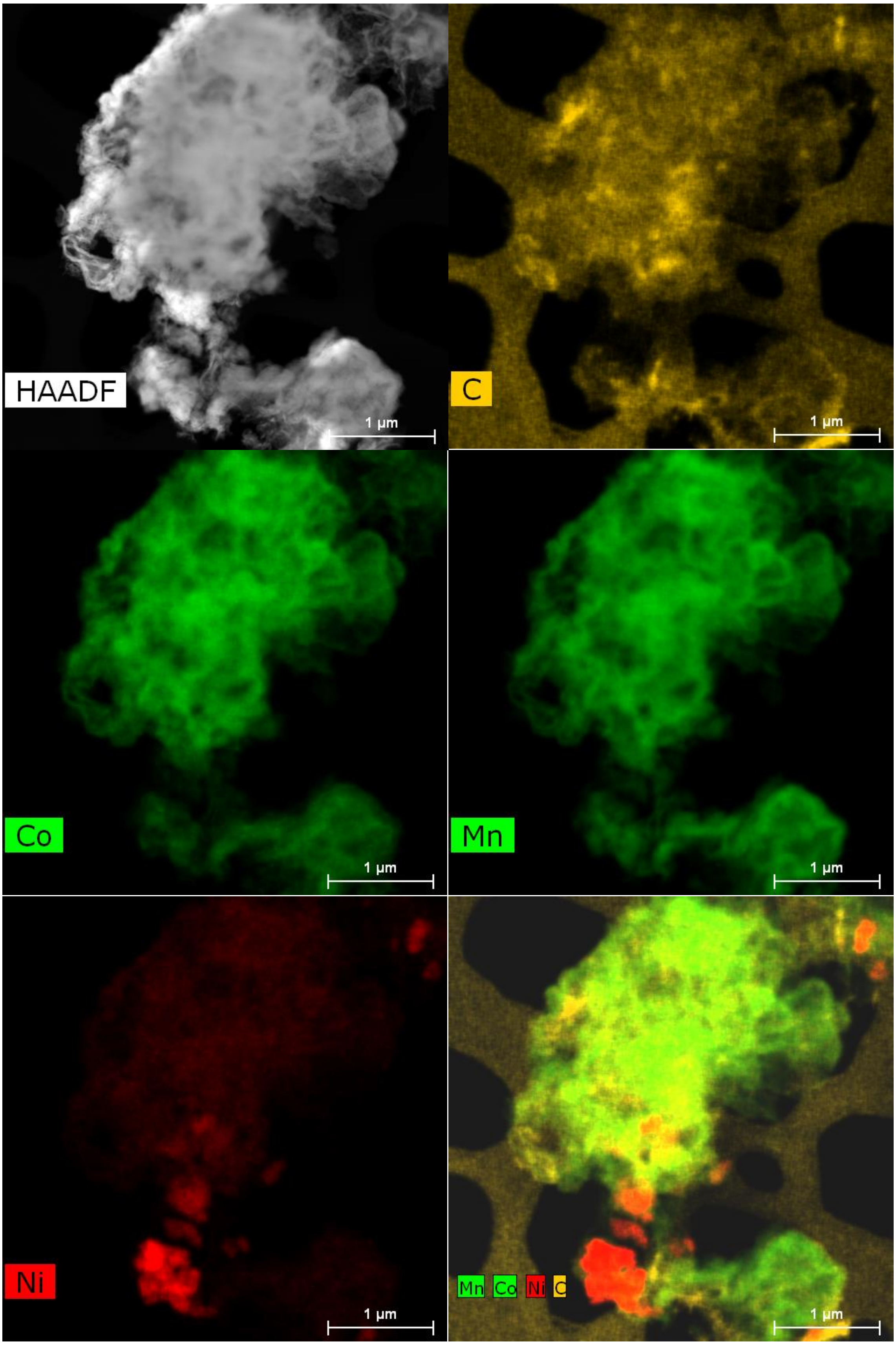

Figure S7. HAADF-STEM image and elemental EDX maps for Mn, Co, Ni and C for the sample **I**. The Ni-containing areas are the Li-rich NMC crystallites.

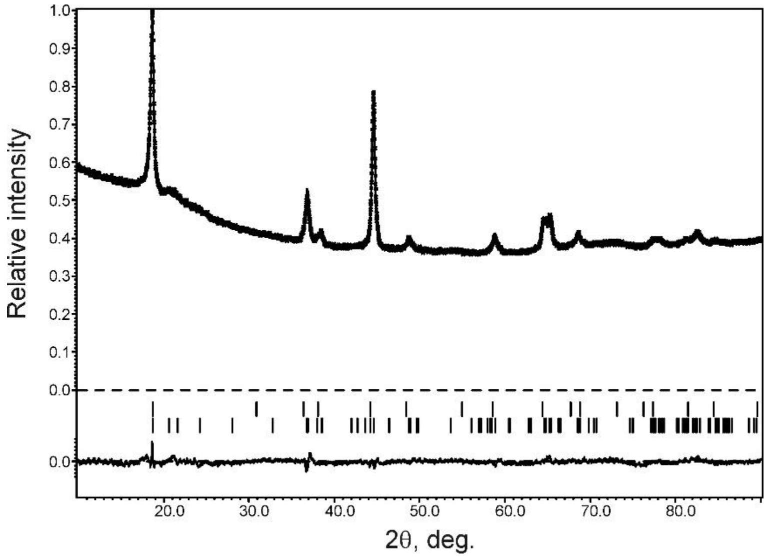

Figure S8. Experimental, calculated and difference PXRD profiles after the Rietveld refinement of the spinel (top reflection row) and $Li_{1.17}Ni_{0.17}Mn_{0.5}Co_{0.17}O_2$ (bottom reflection row) phases in the sample **V**.

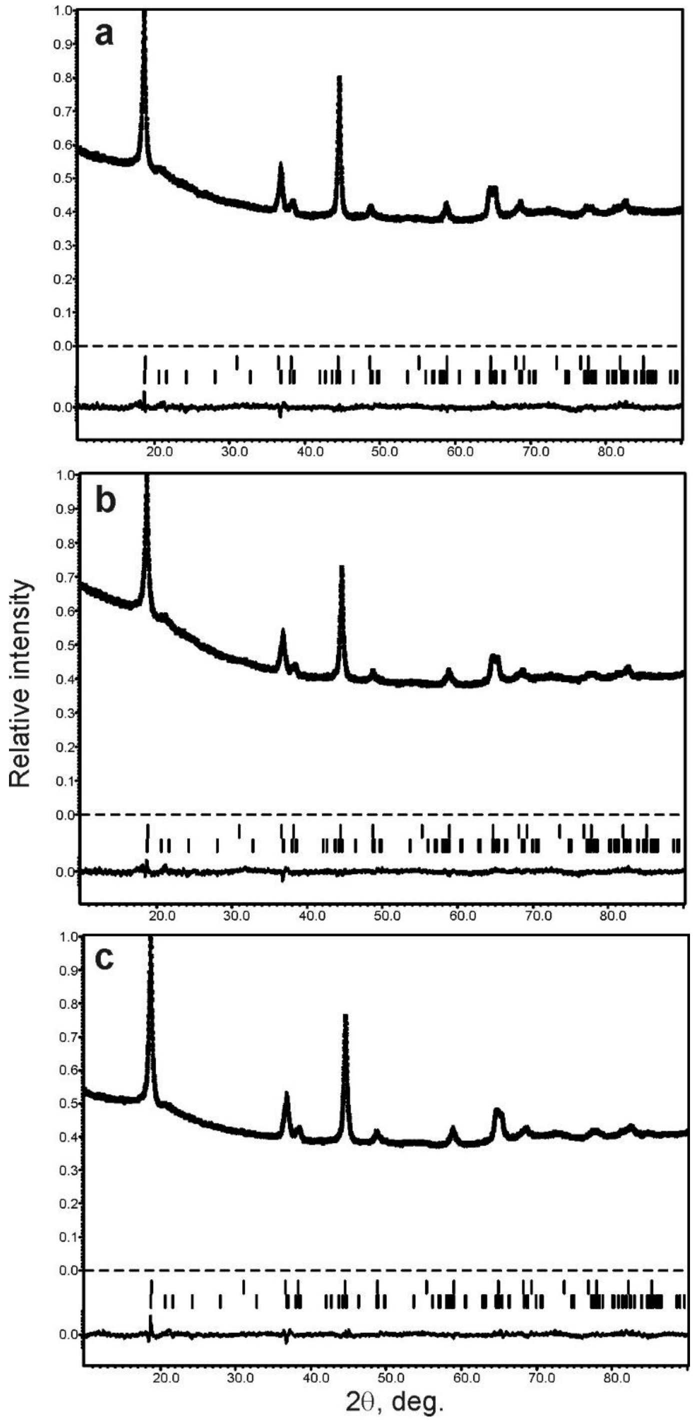

Figure S9. Experimental, calculated and difference PXRD profiles after the Rietveld refinement of the spinel (top reflection row) and $Li_{1.17}Ni_{0.17}Mn_{0.5}Co_{0.17}O_2$ (bottom reflection row) phases in the samples **II** (a), **III** (b) and **IV** (c).

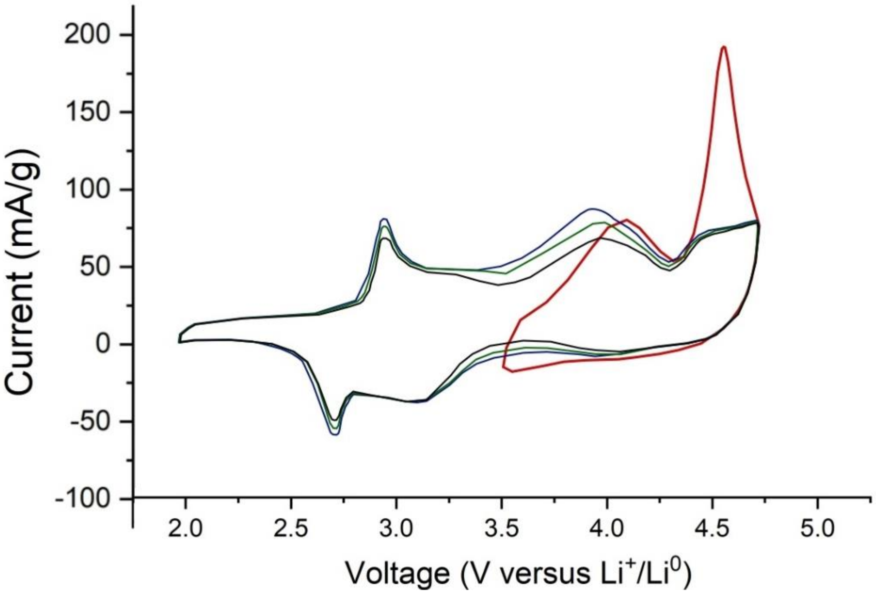

Figure 10. Cyclic voltammetry of the pristine material in the 2.0–4.8V potential range.

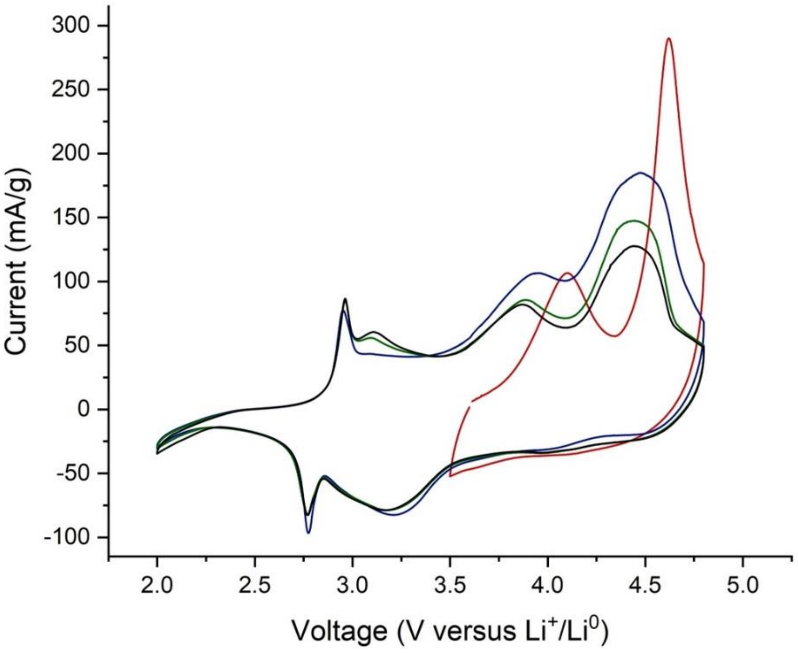

Figure S11. Cyclic voltammetry of the sample II in the 2.0–4.8V potential range.

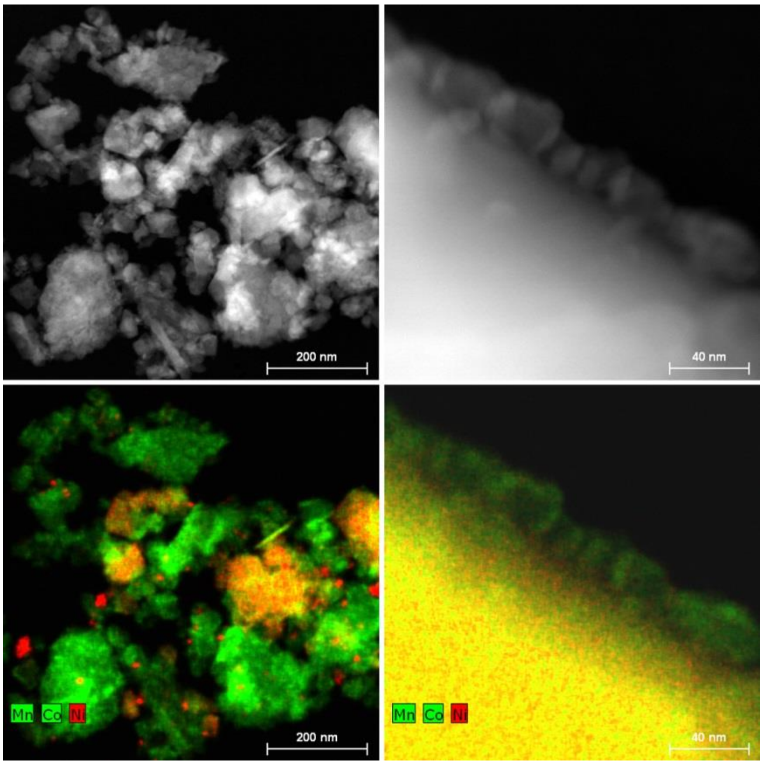

Figure S12. HAADF-STEM images (top) and compositional EDX maps (bottom) demonstrating a presence of spinel as a separate phase (left column) and as nanocrystals covering crystallites of the $Li_{1.17}Ni_{0.17}Mn_{0.5}Co_{0.17}O_2$ phase (right column) for the sample **III** annealed at 400°C with medium precursor concentration. The spinel phase is seen as green areas and nanocrystals at the surface as it does not contain Ni. The Ni-containing areas (red, yellow) are the Li-rich NMC crystals.

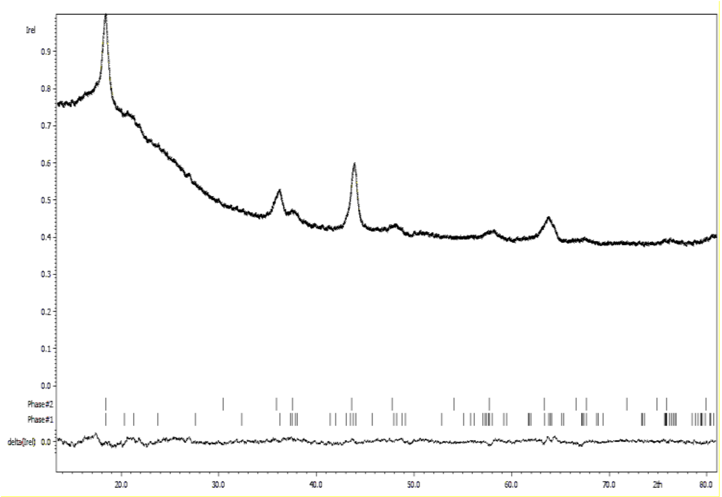

Figure S13. Experimental, calculated and difference PXRD profiles after the Rietveld refinement of the spinel (top reflection row) and $Li_{1.17}Ni_{0.17}Mn_{0.5}Co_{0.17}O_2$ (bottom reflection row) phases in the samples **II** after 25 galvanostatic cycles at C/20.

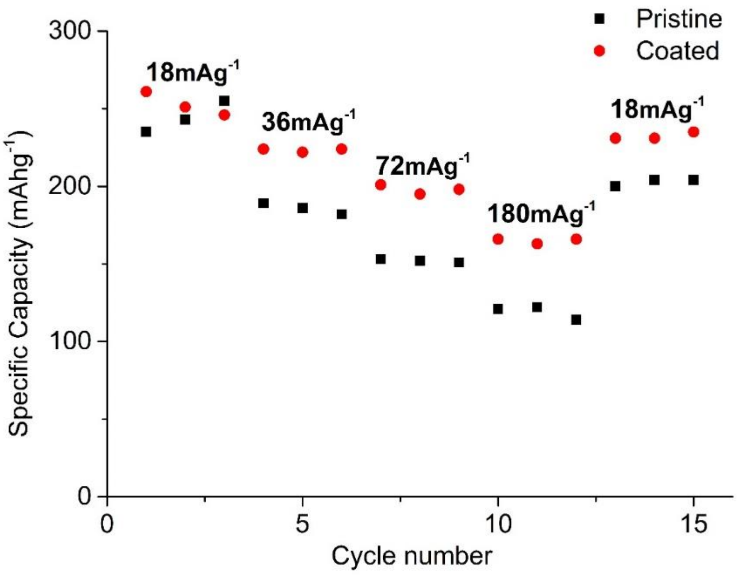

Figure S14. Rate performance of the pristine material (black) and coated (red).

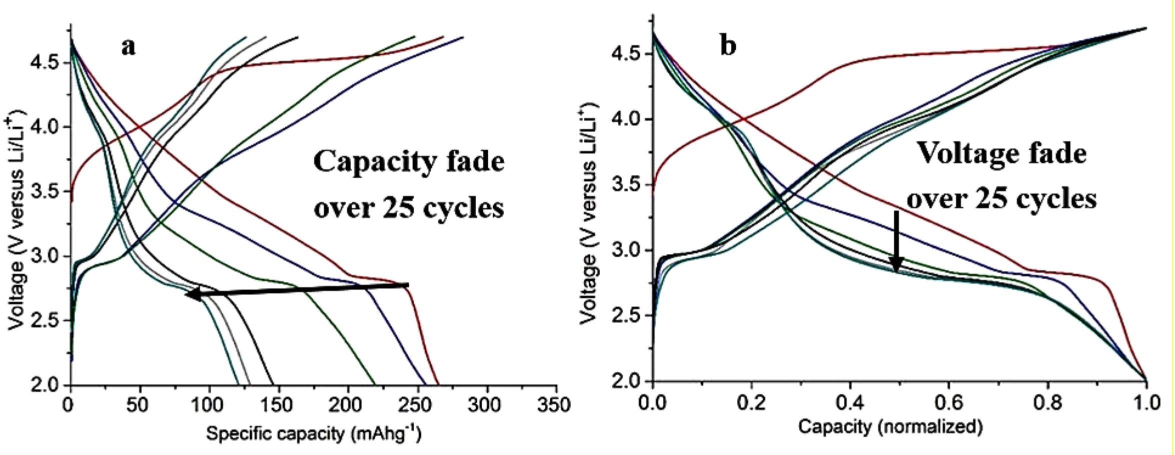

Figure S15. Voltage *vs.* specific capacity plots for the ~24 wt.% spinel-coated sample **III**(a). Voltage *vs.* normalized capacity plots (maximum capacity in each cycle is taken as a unity) for sample **III** (b).

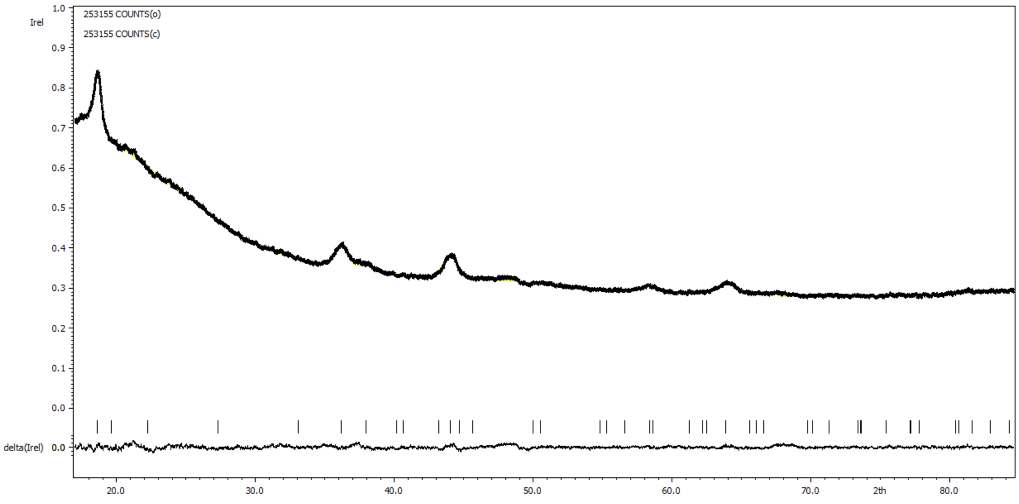

Figure S16. Experimental, calculated and difference PXRD profiles after the leBail refinement of the $Li_{1.17}Ni_{0.17}Mn_{0.5}Co_{0.17}O_2$ phase in the samples **III** after 25 galvanostatic cycles at C/20.

Table S1. Precursor concentration, decomposition temperature, unit cell parameters, phase weight fractions, and the results of EDX analysis of the coated $Li_{1.17}Ni_{0.17}Mn_{0.5}Co_{0.17}O_2$ samples. The core material Li-rich NMC has the monoclinic structure (*C*2/*m*) and the protective spinel coating $LiMn_{1.5}Co_{0.5}O_4$ was identified as a cubic crystal (*Fd*-3*m*).

Sample	Conditions	Phase	*a*,Å	*b*,Å	*c*,Å	β, °	Fraction, wt. %	Mn:Co ratio
**I**	Medium concentration 350 °C	*C*/2*m*	4.930(2)	8.571(3)	5.000(1)	109.51(3)	44.1(7)	
		Fd-3*m*	8.139(1)	-	-	-	55.9(7)	1.4(2):0.6(2)
**II**	Low concentration 400 °C	*C*/2*m*	4.9324(6)	8.578(1)	5.0078(7)	109.34(2)	87.8(5)	
		Fd-3*m*	8.142(4)	-	-	-	12.2(6)	1.66(4):0.34(4)
**III**	Medium concentration 400 °C	*C*/2*m*	4.9334(8)	8.575(1)	5.0070(7)	109.38(2)	77.1(5)	
		Fd-3*m*	8.135(2)	-	-	-	22.9(5)	1.32(7):0.68(7)
**IV**	High concentration 400 °C	*C*/2*m*	4.9270(5)	8.594(1)	5.0006(6)	109.21(1)	75.6(3)	
		Fd-3*m*	8.120(2)	-	-	-	24.4(3)	
**V**	Low concentration 450 °C	*C*/2*m*	4.9303(6)	8.579(1)	5.0080(6)	109.33(2)	97.3(2)	
		Fd-3*m*	8.177(3)	-	-	-	2.7(2)	
